# Attention and Capacity Limits in Perception: A Cellular Metabolism Account

**DOI:** 10.1523/JNEUROSCI.2368-19.2020

**Published:** 2020-08-26

**Authors:** Merit Bruckmaier, Ilias Tachtsidis, Phong Phan, Nilli Lavie

**Affiliations:** ^1^Institute of Cognitive Neuroscience, University College London, London WC1N 3AZ, United Kingdom; ^2^Department of Medical Physics and Biomedical Engineering, University College London, London WC1E 7JE, United Kingdom

**Keywords:** attention, capacity limits in visual perception, cerebral metabolism, inattentional blindness, load theory, oxCCO

## Abstract

Limits on perceptual capacity result in various phenomena of inattentional blindness. Here we propose a neurophysiological account attributing these perceptual capacity limits directly to limits on cerebral cellular metabolism. We hypothesized that overall cerebral energy supply remains constant, regardless of overall mental processing demands; therefore, an attention mechanism is required to regulate limited cellular metabolism levels in line with attended task demands. Increased perceptual load in a task (imposing a greater demand on neural computations) should thus result in increased metabolism underlying attended processing, and reduced metabolism mediating unattended processing. We tested this prediction measuring oxidation states of cytochrome *c* oxidase (oxCCO), an intracellular marker of cellular metabolism. Broadband near-infrared spectroscopy was used to record oxCCO levels from human visual cortex while participants (both sexes) performed a rapid sequential visual search task under either high perceptual load (complex feature-conjunction search) or low load (feature pop-out search). A task-irrelevant, peripheral checkerboard was presented on a random half of trials. Our findings showed that oxCCO levels in visual cortex regions responsive to the attended-task stimuli were increased in high versus low perceptual load, whereas oxCCO levels related to unattended processing were significantly reduced. A negative temporal correlation of these load effects further supported our metabolism trade-off account. These results demonstrate an attentional compensation mechanism that regulates cellular metabolism levels according to processing demands. Moreover, they provide novel evidence for the widely held stipulation that overall cerebral metabolism levels remain constant regardless of mental task demand and establish a neurophysiological account for capacity limits in perception.

**SIGNIFICANCE STATEMENT** We investigated whether capacity limits in perception can be explained by the effects of attention on the allocation of limited cellular metabolic energy for perceptual processing. We measured the oxidation state of cytochrome *c* oxidase, an intracellular measure of metabolism, in human visual cortex during task performance. The results showed increased levels of cellular metabolism associated with attended processing and reduced levels of metabolism underlying unattended processing when the task was more demanding. A temporal correlation between these effects supported an attention-directed metabolism trade-off. These findings support an account for inattentional blindness grounded in cellular biochemistry. They also provide novel evidence for the claim that cerebral processing is limited by a constant energy supply, which thus requires attentional regulation.

## Introduction

Much research has demonstrated the limited nature of perceptual capacity, reporting that, in attention demanding tasks, observers can fail to perceive unattended objects, a phenomenon termed “inattentional blindness” (e.g., Simons and Chabris, 1999; Cartwright-Finch and Lavie, 2007). Neuroimaging research has attributed inattentional blindness to attentional modulations of visual cortex response to unattended stimuli (e.g., Rees et al., 1999).

The level of perceptual load in the task has been shown to be a critical factor in attentional modulations. In tasks involving high perceptual load (e.g., requiring discrimination of feature conjunctions), cortical response to unattended stimuli was found to be smaller compared with low-load tasks (e.g., feature detection). For example, high (vs low) perceptual load in an attended task was shown to result in decreased BOLD signal in V5/MT in response to unattended motion (Rees et al., 1997), in the parahippocampal cortex in response to task-irrelevant images of “places” (Yi et al., 2004), in V1–V4 in response to flickering checkerboard distractors (S. Schwartz et al., 2005; Torralbo et al., 2016), and in V4 and TEO in response to unattended, meaningful objects (e.g., flowers) (Pinsk et al., 2004). This pattern offindings was obtained across a variety of perceptual load manipulations, all known to increase the computational demand on perceptual capacity (Lavie, 2005; Whiteley and Sahani, 2012; Lavie et al., 2014). Behavioral reports also demonstrated the analogous impact of perceptual load on conscious experience (e.g., Carmel et al., 2007; Macdonald and Lavie, 2008; Stolte et al., 2014).

The abundance of studies reporting attentional modulations of the neural response to a variety of stimuli in different cortical regions and across different manipulations of load suggests that they reflect an attentional mechanism, which is required to regulate resources, to accommodate a fundamental, physiological limitation on the overall amount of neural processing. Numerous cellular physiology studies calculating the energy usage of neurons through their ATP consumption have demonstrated that the bioenergetic cost of neural activity is high (Attwell and Laughlin, 2001; Lennie, 2003), primarily because the ion gradients across the cell membrane need to be restored following postsynaptic currents and action potentials. This critically depends on the levels of cellular oxidative metabolism, which supplies the required energy in the form of ATP. Other research has shown that the metabolic energy supply to the brain remains constant regardless of increased mental task demands (Clarke and Sokoloff, 1999). This has led to a widely held premise that cerebral energy supply places a hard limit on mental processing. It follows that increased neural activity (with increased mental-task demand) needs to be balanced out by reductions in cellular metabolism elsewhere. However, while well engrained within theoretical neuroscience, empirical research relating cellular energy limits to limits on mental processing has been rather sparse.

Here we investigated this further, directly testing the impact of perceptual processing demands (load) on the attentional allocation of limited cellular metabolism. We hypothesized thatcellular metabolism levels are flexibly redistributed between attended and unattended stimuli to compensate for changes in demand on the limited metabolic energy available for neural responses. This ensures that metabolic energy is allocated to goal-relevant processing when the overall neural computational demand exceeds the supply, as in conditions of high perceptual load.

In order to provide a straightforward test of this attentional compensatory mechanism that redistributes cellular metabolism according to task demand, a direct assessment of the effect of attention on the underlying cellular metabolism that supplies the required neural energy is necessary. Thus, here we sought to investigate the effects of attention on the distribution of limited cellular metabolic energy to attended versus unattended processing in visual cortex, as assessed with an intracellular marker of metabolism levels. We used broadband near-infrared spectroscopy (BNIRS), which allows us to track the oxidation state of cytochrome *c* oxidase (oxCCO), a mitochondrial enzyme indicative of cellular oxidative metabolism (for review, see Bale et al., 2016), during performance of a selective attention task under different levels of perceptual load.

## Materials and Methods

### 

#### BNIRS methodology

The oxCCO signal measured with BNIRS provides an intracellular marker of oxidative metabolism levels. Increases in energy requirements because of neuronal activation are largely covered by an upregulation of oxidative phosphorylation whereby energy in the form of ∼30 ATP molecules (commonly known as the molecular unit of currency for intracellular transfer of energy) per glucose molecule are produced (Attwell et al., 2010; Lin et al., 2010). CCO is the final electron acceptor of the electron transport chain in the mitochondria where oxidative phosphorylation takes place. Since its concentration does not change over relatively short time periods (e.g., hours), the ratio between oxidized and reduced CCO can be used to assess changes to the level of cellular metabolism. BNIRS can measure the oxCCO signal by using the full light spectrum in the range of 780–900 nm (Arifler et al., 2015). Conventional functional near-infrared spectroscopy systems, in contrast, have just 2–3 wavelengths of light and thus can only be used to measure concentration changes in oxygenated hemoglobin (HbO_2_) and deoxygenated hemoglobin (HHb) in the blood vessels surrounding the brain areas of interest. The intracellular BNIRS measure of oxCCO has been validated both in animal and human studies, for example, demonstrating its correlation with phosphorous magnetic resonance spectroscopy (^31^P MRS) measures of nucleotide triphosphate levels (which is mainly ATP) (Peeters-Scholte et al., 2004; Bainbridge et al., 2014; Kaynezhad et al., 2019) and measures of the lactate/pyruvate ratio, a marker of aerobic metabolism (i.e., mitochondrial ATP synthesis), as obtained with microdialysis (Tisdall et al., 2008; for review, see Bale et al., 2016).

In the present study, we used a multichannel BNIRS system, which has been developed to specifically measure oxCCO (e.g., Phan et al., 2016) and has been shown to successfully isolate its signal (based on the absorption characteristics of oxCCO, which has a broad peak at 830 nm) from other chromophores (HHb and HbO_2_), as described here (Siddiqui et al., 2018). The instrument has 4 source and 10 detector fibers (optodes) and a sampling rate of 1 s. The detectors were arranged in rows of five with the four sources between them (source detector separation was 30 mm), resulting in 16 measurement channels. The array was fitted horizontally in a custom-designed optode holder, the center of which was placed 4 cm above the inion. All optode positions were digitized using a Patriot Digitizer (Polhemus), and the inion, nasion, left, and right preauricular points, O1, O2, and vertex served as reference points (based on 10/20 electrode placement system). To ensure that the positions of the channels matched between participants, the digitized locations were converted to MNI coordinates using NIRS SPM (Ye et al., 2009).

#### Experiment 1: experimental design and statistical analysis

In Experiment 1, we first sought to establish whether the metabolismlevels associated with unattended processing are affected by the level of perceptual load in the attended task. To that purpose, we have used a well-established perceptual load manipulation, which includes a rapid, serial visual search task that is accompanied by a task-irrelevant, flickering checkerboard in the periphery on half of the trials (S. Schwartz et al., 2005; Carmel et al., 2011; Ohta et al., 2012). We examined the effects of perceptual load in the attended task on the levels of metabolism specifically associated with the unattended, peripheral checkerboard. Since the purpose of Experiment 1 was to investigate whether perceptual load can modulate the levels of metabolism associated with the processing of unattended stimuli, the size of the checkerboard was maximized (relative to the attended task stimuli) to ensure that we would be able to measure a strong signal associated with unattended stimulus processing in the low-load condition, as well as a modulation of this response under high perceptual load.

##### Participants

Sixteen participants (11 female, age range 18-34 years) took part in Experiment 1. Since this is the first study using BNIRS to measure effects of attention on visual processing, no formal sample size calculations could be conducted. We therefore used a sample size that is comparable to studies using other neuroimaging techniques, looking at similar effects of perceptual load on cortical processing (e.g., S. Schwartz et al., 2005, 16 participants; Molloy et al., 2015, 14 participants; Torralbo et al., 2016, 18 participants). A sensitivity analysis on the results obtained in this experiment, using MorePower (Campbell and Thompson, 2012), confirmed that this sample size was sufficient to detect effects of a size η*_p_*^2^ ≥ 0.37 with a power of 80%. All participants had normal or corrected-to-normal vision and normal color vision. This research was approved by the UCL research ethics committee, and written, informed consent was obtained from all participants before data collection.

##### Task and stimuli

**Figure 1.**
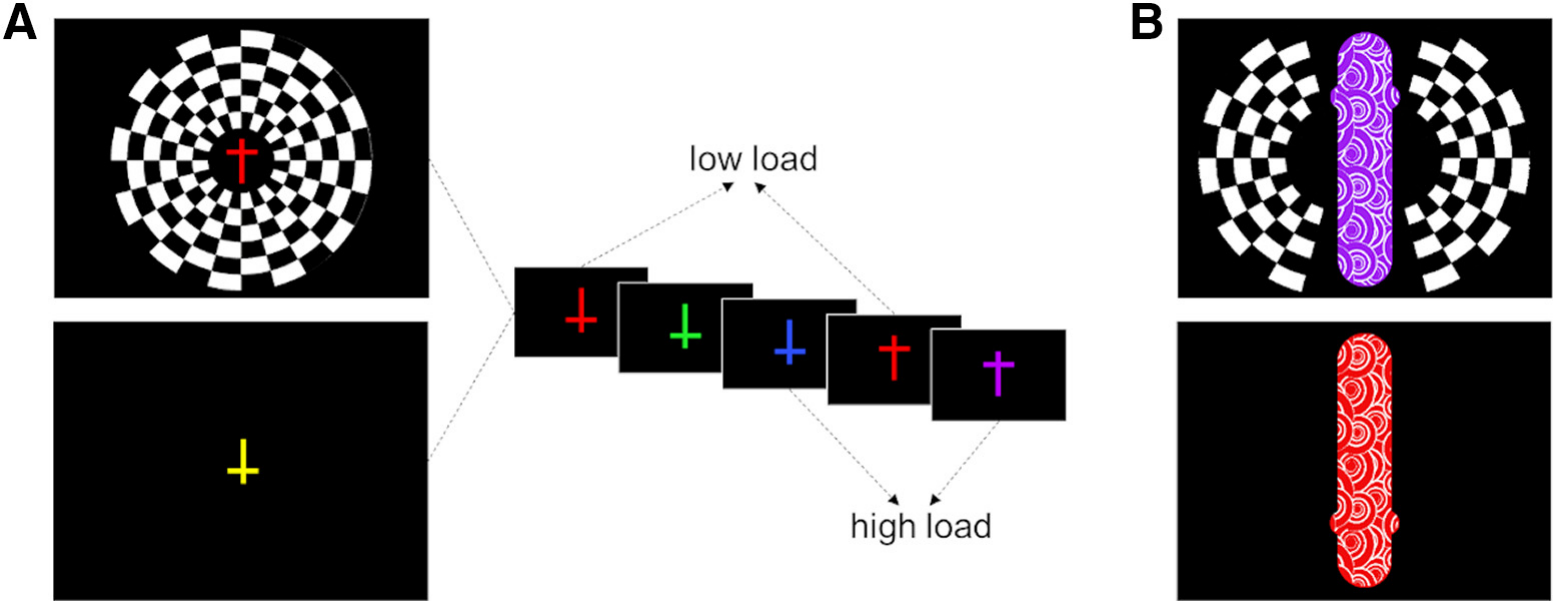
Experimental design. ***A***, Experimental task in Experiment 1. Participants saw a stream of colored crosses and had to respond to feature targets in low load (any red cross) or conjunction targets in high load (upright purple or inverted blue crosses). A flickering, radial checkerboard was present on half of the RSVP streams. ***B***, Experimental task in Experiment 2. The size of the crosses was increased, and a white pattern was added to increase the strength of the response in visual cortex. Images are not to scale.

The experiment took place in a darkened room to minimize external light interfering with the BNIRS system. We presented the experiments with MATLAB Cogent Graphics tool box. The attended task display consisted of a series of crosses (each 0.08° × 0.06° of visual angle), colored either blue (0, 115, 255), green (0, 255, 0), yellow (255, 255, 0), purple (160, 32, 240), red (255, 0, 0), or brown (156, 102, 31), and oriented either upright or inverted. These stimuli were presented rapidly in the center of the computer screen on a black background (Fig. 1) (see S. Schwartz et al., 2005; Carmel et al., 2011). On half of the streams, a black-and-white, radial checkerboard, which was flickering at a frequency of 7 Hz, was present in the periphery of the visual field (extending 17° of visual angle from the center of the screen, leaving out a circle with a radius of 0.7° in the center where the targets were presented). Participants were instructed to ignore the checkerboard stimulus, if present. Their task was to detect prespecified “target” crosses by pressing the “0” key on the number pad of the computer keyboard. In the low-load condition, the targets were determined by color alone (any red crosses), whereas in the high-load condition targets were determined by a conjunction of color and orientation (upright purple and inverted blue crosses).

Each 32 item stream started with a fixation cross present for 1000 ms at the center of the screen, followed by the presentation of 32 crosses (250 ms), each followed by a 500 ms interstimulus interval. Each stream contained 4 targets (12.5% of stimuli) that were presented randomly in any temporal stream positions, except for the first, with the constraint that no two targets could appear on successive presentations. The time window of 750 ms from the onset of a target has previously been shown to provide sufficient time for typical responses to be made before the next stimulus appeared (e.g., Carmel et al., 2011). However, the constraint that no two targets could appear in succession allowed us to accept any target detection response made within the 1500 ms time window from target onset (the minimal time between two potential targets) as correct. The target stimulus was equally likely to be in either orientation in the low-load or color/orientation combination in the high-load condition (sampled randomly with replacement). Apart from excluding the target color (in the low load) and target color/orientation combination (in the high load), all colors and color/orientation combinations were equiprobable for each of the nontarget stimuli (sampled randomly with replacement), with the exception that the opposite feature combination of those defining the targets in the high-load condition(i.e., upright blue and inverted purple) were twice as likely as any other nontarget color/orientation combination. To match the streams across the load conditions, these opposite combinations were also twice as likely in the low-load streams. The visual stimulation was thus the same in both load conditions, and load was varied just through the task instructions, which required a different amount of perceptual processing for the same stimulus stream. Participants completed 56 streams, each consisting of 32 items, lasting for 25 s, and followed by a 25 s break, during which participants received automated feedback on their performance. Five seconds before the next stream, instructions indicating the new targets appeared on the screen. Participants started with two short practice streams (one per load condition, always starting with low load). The experimental streams were interleaved in an ABBABAAB pattern with respect to the load condition.

##### Data preprocessing

In order to convert the measured attenuation changes across the wavelengths between 780 and 900 nm into concentration changes of the chromophores (HHb, HbO_2_, oxCCO), we applied the UCLn algorithm using the Modified Beer-Lambert law assuming a differential pathlength factor of 6.26 and its wavelength dependence (Phan et al., 2016). Next, the concentration changes of each chromophore were bandpass filtered to remove physiological noise (e.g., Mayer waves) using a fifth-order Butterworth filter with cutoff frequencies of 0.01 and 0.08 Hz. Streams were excluded from analysis if motion artifacts were present or if the error rate was particularly high (≥75%), potentially indicating that the participant was responding to the wrong targets. This resulted in 3.46% of trials in total removed in Experiment 1 and 6.05% of trials removed in Experiment 2. For each participant and channel, the data were then prepared by averaging across the RSVP streams for each of the four conditions (high/low load × checkerboard present/absent), using the first second of each RSVP stream as the baseline by subtracting it from the activity in the rest of the trial.

The converted MNI coordinates indicated that our channel positions were located across Brodmann areas (BAs) 17, 18, and 19, commonly referred to as striate cortex and visual association areas. Based on their MNI coordinates, measurement channels were allocated individually for each participant (Ye et al., 2009) to the following cortical regions: left and right BA19, left and right BA18, and BA17 (for average coordinates and allocation of each channel in Experiment 1 and 2, see Tables 1 and 2, respectively). This step reduced the number of statistical comparisons compared with the channel level and therefore lowered the risk of false positives.

**Table 1.** Average channel positions and BA allocations in Experiment 1*^a^*

Channel	MNI coordinates			BA	Probability
	*x*	*y*	*z*		
1	−54.73	−76.19	12.31		
				19	0.63
				37	0.21
				39	0.15
2	−43.38	−89.02	16.77		
				18	0.12
				19	0.88
3	−51.31	−80.27	−6.40		
				19	0.90
				37	0.10
4	−42.29	−92.29	−2.92		
				18	0.56
				19	0.44
5	−28.88	−97.85	18.40		
				17	0.28
				18	0.51
				19	0.21
6	−12.98	−103.90	19.06		
				17	0.83
				18	0.17
7	−28.25	−102.60	−0.15		
				17	0.62
				18	0.38
8	−13.98	−107.77	1.63		
				17	1.00
9	7.17	−100.92	17.15		
				17	0.74
				18	0.26
10	24.00	−101.54	15.94		
				17	0.76
				18	0.24
11	6.40	−102.69	1.50		
				17	1.00
12	22.79	−104.94	−1.96		
				17	0.86
				18	0.14
13	39.23	−92.13	13.04		
				17	0.05
				18	0.49
				19	0.46
14	52.94	−79.10	9.52		
				19	0.79
				37	0.15
				39	0.06
15	37.50	−95.50	−6.00		
				18	0.93
				19	0.07
16	48.52	−83.44	−10.63		
				18	0.03
				19	0.97

*^a^*Overview of group-averaged MNI coordinates and assignment to BAs.

**Table 2.** Average channel positions and BA allocations in Experiment 2*^a^*

Channel	MNI coordinates			BA	Probability
	*x*	*y*	*z*		
1	−56.94	−70.48	22.33		
				19	0.10
				37	0.08
				39	0.82
2	−45.39	−84.09	25.93		
				19	0.74
				39	0.26
3	−54.56	−76.91	0.13		
				19	0.66
				37	0.34
					
4	−44.83	−89.89	3.28		
				18	0.39
				19	0.61
5	−30.20	−93.74	26.07		
				18	0.50
				19	0.50
6	−14.20	−99.93	25.78		
				17	0.35
				18	0.65
7	−30.57	−100.80	3.91		
				17	0.50
				18	0.50
8	−14.76	−107.39	4.43		
				17	1.00
9	7.52	−97.67	24.81		
				17	0.21
				18	0.75
				19	0.03
10	24.81	−95.87	26.31		
				17	0.08
				18	0.89
				19	0.03
11	7.00	−103.59	4.57		
				17	1.00
12	24.67	−103.98	4.98		
				17	0.93
				18	0.07
13	40.96	−86.83	25.76		
				18	0.01
				19	0.95
				39	0.04
14	53.89	−74.43	23.89		
				19	0.13
				37	0.02
				39	0.85
15	39.94	−93.46	3.63		
				17	0.03
				18	0.79
				19	0.18
					
16	52.24	−80.74	1.83		
				19	0.92
				37	0.08

*^a^*Overview of group-averaged MNI coordinates and assignment to BAs.

##### Statistical analysis

In both Experiments 1 and 2, analyses of the oxCCO were based on the mean oxCCO signal in each of the conditions for each participant across the 25 s task period. In all analyses of both the oxCCO and the behavioral data, the outlier exclusion criterion was based on responses that are >2.5 SDs from the group mean. This resulted in the exclusion of 1 participant in each of the experiments. Behavioral responses were compared using pairwise, two-tailed *t* tests comparing response times, hit rates, and false alarm rates between high- versus low-load conditions. The main oxCCO analysis used a 2 × 2 within-subject ANOVA to investigate the effects of distractor presence (present vs absent) and perceptual load (high vs low). Statistical significance is reported using an α level of 0.05 with false discovery rate (FDR) correction (Benjamini and Hochberg, 1995) for multiple comparisons across the five cortical regions.

#### Experiment 2: experimental design and statistical analysis

In Experiment 2, we investigated whether the modulation of the metabolism associated with unattended processing in Experiment 1 was the result of a load-induced trade-off, as suggested by previous functional imaging experiments (Pinsk et al., 2004; Torralbo et al., 2016). We therefore examined whether the reduction of metabolic energy associated with unattended processing by high perceptual load was accompanied by a simultaneous increase in metabolism underlying attended processing. To that purpose, we have modified the task used to increase its sensitivity to reveal the effects of load on the attended stimuli, as follows: The size of the central crosses was substantially increased to produce a greater oxCCO signal. Furthermore, a white pattern of swirls was overlaid over each cross to increase the changes in local contrast and therefore to further increase the extent to which the attended stimuli activated striate and extrastriate visual cortex regions (Fig. 1).

##### Participants

Power analysis using MorePower (Campbell and Thompson, 2012), based on the effect sizes observed in Experiment 1, indicated that a sample of 12-18 participants (depending on which BA was used for the calculation) is required to detect the load effect on unattended processing with α = 0.05 and 80% power. We collected data from 18 participants (15 female, age range 20-38 years), which satisfies the more conservative estimate of sample size, all with normal or corrected-to-normal vision and normal color vision. One participant participated in both experiments; the rest were naive.

##### Task and stimuli

In order to establish load effects on metabolism associated with attended processing (in addition to unattended processing), we increased the size of the central crosses (vertical bar: height: 23.7°, width: 5.1°; horizontal bar: height: 2.1°, width: 6.7°; midline of horizontal bar was placed at 6.1° from the [top/bottom] end of the vertical bar) and overlaid them with a white pattern of swirls to increase the local contrast and therefore the extent to which they activate early visual cortex regions (Fig. 1). The distractors were two flickering, radial checkerboard segments on either side of the central task (147° of arc, with a radius extending 12.8° of visual angle from the center of the screen, leaving a circle of 5.7° in radius free in the center). Thus, both the attended and unattended stimuli should now elicit a measurable signal that allows us to track any modulation induced by changes to perceptual load. Since the stimuli were far larger now, we ensured that participants would still fixate at the center of the stimuli to process both the bottom and the top horizontal cross bars, and avoid a strategy of judging the horizontal bar location not just by its presence but also from its absence at one fixated position (either the top or the bottom of each cross), by including nontarget stimuli that consisted only of the vertical bar of the cross on a random third of stimulus presentations (colors selected in random from the stimulus set regardless of whether nontarget or target colors). Subjects were instructed to withhold responses to these stimuli (including when presented in the target color in the low-load conditions). All other details remained the same as in Experiment 1.

##### Statistical analysis

Following the same exclusion criteria as for Experiment 1, 1 subject was excluded from analysis in Experiment 2. Areas showing significant effects in Experiment 1 served as ROI (bilaterally) for the within-subject 2 × 2 (load × distractor conditions) ANOVAs in Experiment 2, while FDR correction was applied to all other analyses (including the attended processing analysis) since the regions for these have not been previously established. For this reason, the simple main effects concerning attended processing (distractor-absent conditions) were not reported in the ROI-based 2 × 2 ANOVAs (of distractor condition × load). In addition to the same ANOVAs as those run in Experiment 1, we also performed pairwise *t* tests to compare the mean oxCCO response during the 25 s task period in distractor-absent trials in high versus low perceptual load, which reflects the activity associated specifically with the processing of the attended task stimuli (without distractor stimuli).

In Experiment 2, we also analyzed the temporal correlation of the load effect on attended processing (High Absent – Low Absent) and unattended processing ((High Present – High Absent) – (Low Present – Low Absent)), during the 25 s task period. The group mean for each second-by-second time point in each time series was computed, following a trial-splitting procedure that was conducted to ensure that the data entered into each participant's time series did not include overlapping trials (since the distractor-absent condition was used for both the attended and unattended signal), as follows. We split the distractor-absent raw data randomly into two halves for each participant: One half was used for the attended time series and the other used for the unattended time series, before the two time series got averaged across all participants to provide the group mean for each second-by-second data point in the two time series. A Kolmogorov–Smirnov test verified that the data were normally distributed and therefore suitable for a Pearson correlation. To avoid sample bias from the random splitting of the data, we repeated the random data split 1000 times, and a Pearson correlation was conducted on the attended versus unattended time series in each of the 1000 samples. We note that this correlation analysis treats subjects as fixed rather than random effects, and thus only allows inferences about the specific sample, not the whole population. A replication of this analysis with a larger sample (that allows for a correlation analysis that treats subjects as random effects) is important to further support the temporal “push-and-pull” nature of the resource trade-off we have observed.

In order to assess significance of the mean *r*, we used a permutation test with 10,000 permutations, using the same 1000 samples but with randomly assigned condition labels (to each participant's time series in each sample). A significance threshold of 95% was then used for the comparison of the mean *r* value obtained from the correctly labeled data with the distribution of 10,000 mean *r* values from the random permutations (i.e., to be considered significant, the mean *r* value from the correctly labeled data had to be >9500 of the mean *r* values obtained from the data with randomly shuffled condition labels). The use of the permutation analysis controls for any effects of dependence of data points within each subject in the correlated time series (e.g., autocorrelation), since these are equally present in the time series with permuted condition labels.

## Results

### Experiment 1

#### Behavioral data

Behavioral results (Table 3) confirmed that higher perceptual load in the attended task increased task reaction times (*t*_(14)_ = 18.36, *p* < 0.001, *d* = 5.19), reduced hit rates (*t*_(14)_ = −3.41, *p* = 0.004, *d* = −0.90), and increased the number of false alarms (*t*_(14)_ = 3.76, *p* = 0.002, *d* = 1.06), thus confirming the efficacy of the perceptual load manipulation.

**Table 3.** Behavioral results from Experiment 1*^a^*

	Low load	High load
Reaction time (ms)	491 (48)	619 (40)
Hit rate (%)	99.02 (1.76)	95.90 (4.27)
False alarm rate (%)	0.03 (0.48)	4.95 (5.00)

*^a^*Data are task performance mean (SD).

#### oxCCO data

**Figure 2.**
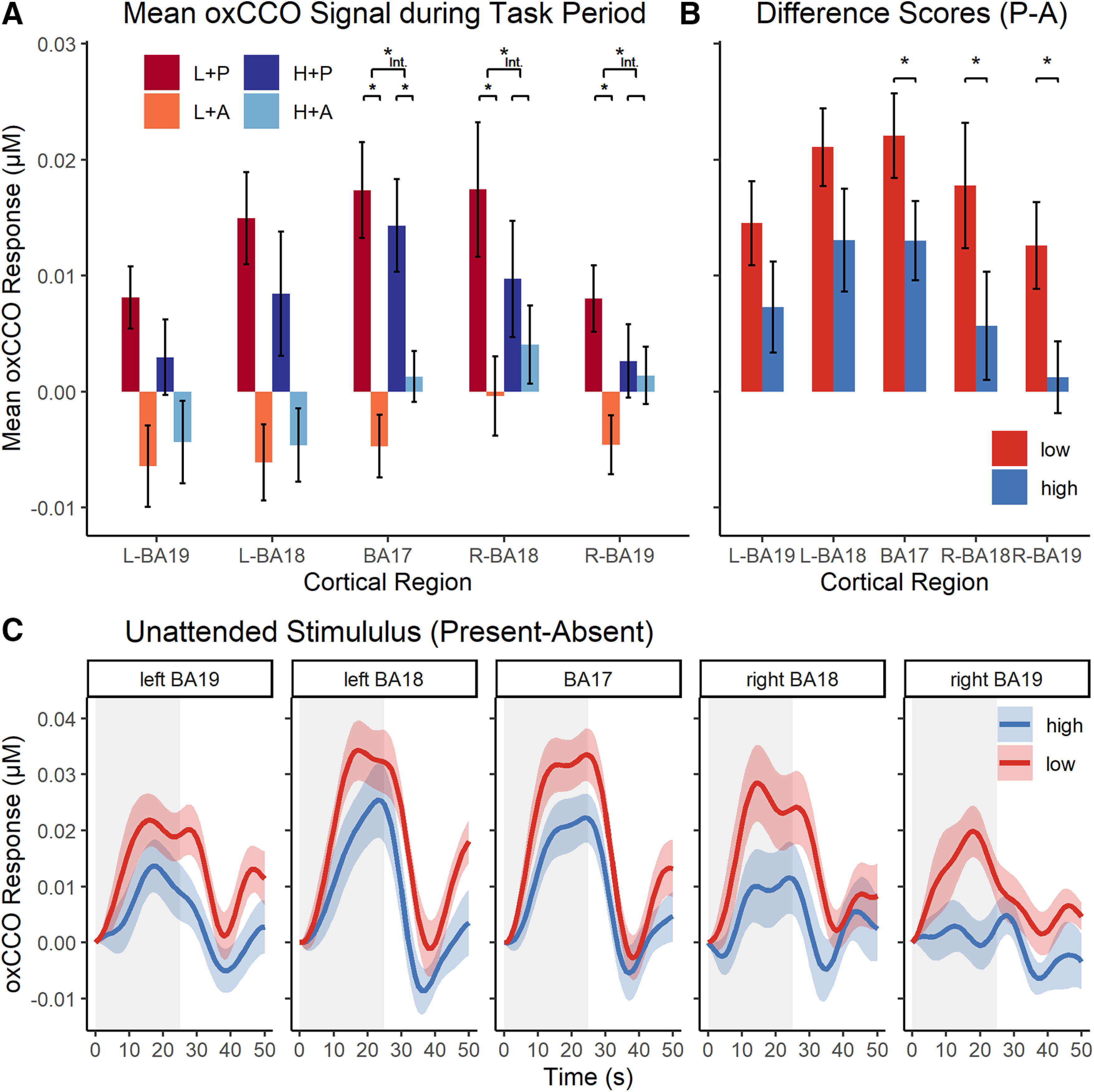
oxCCO concentration changes in Experiment 1. ***A***, Mean (±SEM) oxCCO signal per condition (high/low load × checkerboard present/absent) across the task period (25 s) for all investigated regions. ***B***, Difference scores (distractor-present minus distractor-absent conditions) of the mean oxCCO signals (±SEM) by load, illustrating the nature of interactions in ***A***. ***C***, Time series of the group-averaged oxCCO signal related to the presence (minus absence) of the unattended stimulus. Gray, shaded areas represent the task period (25 s, followed by a 25 s rest period). Colored areas along the graphs represent the SEM. *p < 0.05. Int., Interaction.

The oxCCO results are shown in Figure 2. As can be seen in Figure 2*A*, the mean oxCCO response during the task period was larger when the distractor was present than when it was absent, as was confirmed with a main effect of distractor presence in all BAs (left BA19: *F*_(1,14)_ = 12.53 *p*_FDR_ = 0.005, η*_p_*^2^ = 0.47; left BA18: *F*_(1,14)_ = 24.61, *p*_FDR_ < 0.001, η*_p_*^2^ = 0.65; BA17: *F*_(1,14)_ = 27.85, *p*_FDR_ < 0.001, η*_p_*^2^ = 0.67; right BA18: *F*_(1,14)_ = 6.28, *p*_FDR_ = 0.031, η*_p_*^2^ = 0.31; right BA19: *F*_(1,14)_ = 4.81, *p*_FDR_ = 0.046, η*_p_*^2^ = 0.26). Importantly, Figure 2*B*, *C* also shows that the oxCCO signal associated with the distractor presence (vs absence) was reduced in the high-load (compared with low-load) conditions, as predicted. This interaction effect (of load and distractor conditions) was significant in BA17 (*F*_(1,14)_ = 9.10, *p*_FDR_ = 0.023, η*_p_*^2^ = 0.39), right BA18 (*F*_(1,14)_ = 6.84, *p*_FDR_ = 0.034, η*_p_*^2^ = 0.32), and right BA19 (*F*_(1,14)_ = 12.25, *p*_FDR_ = 0.018, η*_p_*^2^ = 0.47). Indeed, in both right BA18 and right BA19, the distractor effect was only significant in the low-load conditions (right BA18: *t*_(14)_ = 3.18, *p* = 0.007; right BA19: *t*_(14)_ = 3.26, *p* = 0.006), but not in the high-load conditions (right BA18: *t*_(14)_ = 1.18, *p* = 0.259; right BA19: *t*_(14)_ = 0.39, *p* = 0.704), while in BA 17 it remained significant in both load conditions (low load: *t*_(14)_ = 5.87, *p* < 0.001; high load: *t*_(14)_ = 3.69, *p* = 0.002). Similar trends of the load-distractor interaction did not reach significance in left BA18 (*F*_(1,14)_ = 3.39,*p*_FDR_ = 0.110) and left BA19 (*F*_(1,14)_ = 2.25, *p*_FDR_ = 0.156). There was no main effect of perceptual load in any area (all *p*_FDR_ > 0.813), as might be expected given the terminative nature of the interaction. Finally, a comparison of the baselines used in the low load and high load revealed no significant difference (mean difference ≤ 0.0042 μm, all *p*_FDR_ > 0.655).

### Experiment 2

In order to further establish whether the observed reduction of the oxCCO signal related to unattended processing in Experiment 1 results from a resource trade-off relationship with the attended processing, in Experiment 2 we compared the impact of perceptual load on cellular metabolism levels in attended versus unattended processing using modified task stimuli better suited to reveal BNIRS signals from both types of stimuli.

#### Behavioral data

As in Experiment 1, behavioral results (Table 4) showed that high perceptual load increased reaction times (*t*_(16)_ = 16.08, *p* < 0.001, *d* = 3.90), reduced hit rates (*t*_(16)_ = −2.83, *p* = 0.012,*d* = −0.69), and increased false alarm rates (*t*_(16)_ = 2.17, *p* = 0.046, *d* = 0.53), thus successfully manipulating task demand.

**Table 4.** Behavioral results from Experiment 2*^a^*

	Low load	High load
Reaction time (ms)	510 (56)	599 (67)
Hit rate (%)	98.79 (1.61)	95.92 (4.48)
False alarm rate (%)	1.16 (1.07)	6.17 (10.11)

^a^>Data are task performance mean (SD).

#### oxCCO data

**Figure 3.**
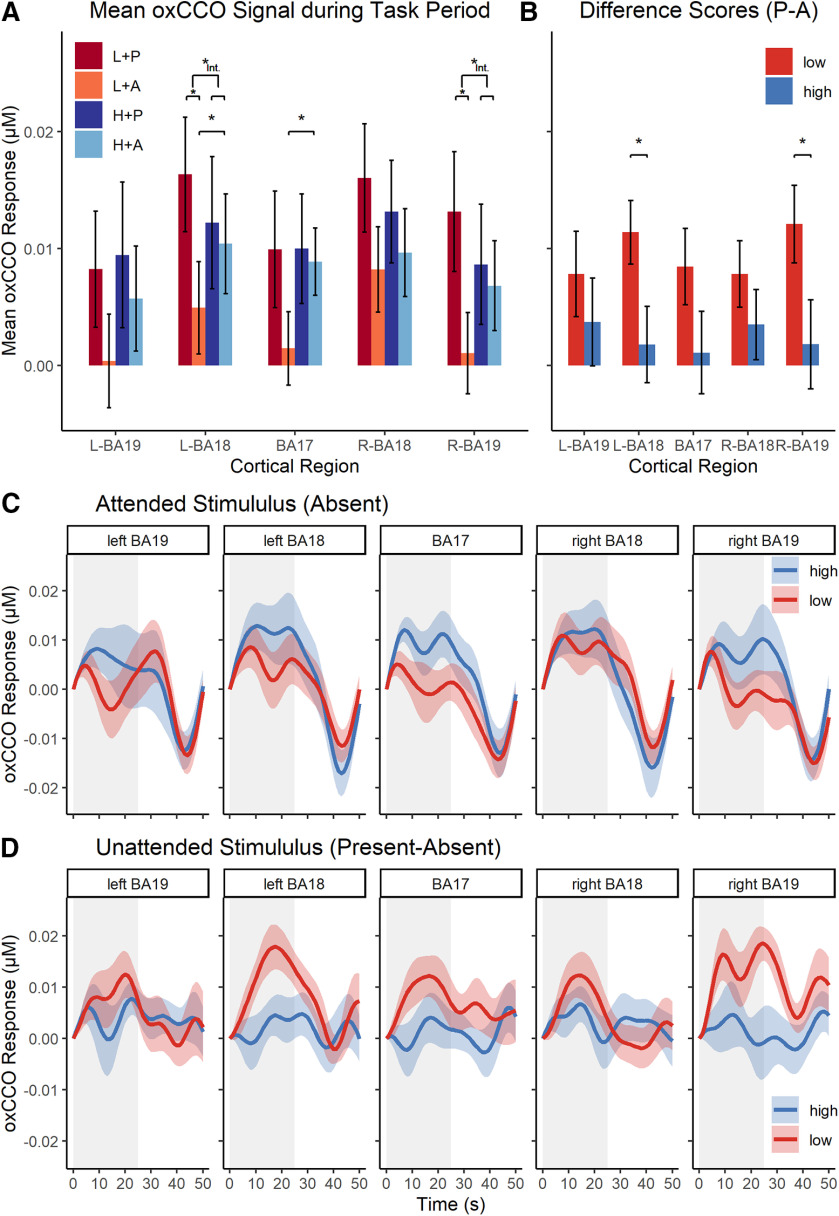
oxCCO concentration changes in Experiment 2. ***A***, Mean (±SEM) oxCCO signal per condition (high/low load × checkerboard present/absent) during the task period (25 s) across all investigated regions. ***B***, Difference scores of the mean oxCCO signals (±SEM) in the distractor-present minus distractor-absent conditions plotted as a function of load, illustrating the nature of interactions in ***A***. ***C***, ***D***, Time series of the group-averaged oxCCO signal related to the attended (***C***, distractor-absent conditions only) and unattended (***D***, difference score of distractor-present minus distractor-absent trials) stimuli. Gray, shaded areas represent the task period (25 s, followed by a 25 s rest period). Colored areas along the graphs represent the SEM. *p < 0.05. Int., Interaction.

The oxCCO results of Experiment 2 are shown in Figure 3. As can be seen in the figure, the effect of perceptual load on distractor processing found in Experiment 1 was replicated in Experiment 2. Specifically, Figure 3*A* shows that the mean oxCCO response during the task period was larger when the distractor was present compared with when it was absent, and this was reflected in the significant main effects of distractor presence (vs absence) in left BA18 (*F*_(1,16)_ = 6.68, *p* = 0.012, η*_p_*^2^ = 0.29), right BA18 (*F*_(1,16)_ = 5.24, *p* = 0.036, η*_p_*^2^ = 0.25), and right BA19 (*F*_(1,16)_ = 6.10, *p* = 0.025, η*_p_*^2^ = 0.28). Similar trends did not reach significance in left BA19 (*F*_(1,16)_ = 3.77, *p* = 0.070) and BA17 (*F*_(1,16)_ = 2.84, *p* = 0.111). Importantly, Figure 3*B*, *D* shows that, as in Experiment 1, the oxCCO signal change associated with the presence (vs absence) of the distractor was reduced in the high (vs low) perceptual load conditions, and this was confirmed by significant interactions between load and distractor presence in left BA18 (*F*_(1,16)_ = 7.51, *p* = 0.015, η*_p_*^2^ = 0.32) and right BA19 (*F*_(1,16)_ = 4.74, *p* = 0.045, η*_p_*^2^ = 0.23). In both areas, the distractor presence (vs absence) effect was significant in the low-load (left BA18: *t*_(16)_ = 4.07, *p* = 0.001; right BA19: *t*_(16)_ = 3.54, *p* = 0.003) but not the high-load conditions (left BA18: *t*_(16)_ = 0.53, *p* = 0.600; right BA19: *t*_(16)_ = 0.47, *p* = 0.648). Similar interaction trends did not reach significance in the other BAs (all *F* < 3.22, *p* > 0.092). There were no main effects of load in any areas apart from BA17, which showed a significantly increased signal in the high-load compared with the low-load conditions (*F*_(1,16)_ = 4.91, *p* = 0.041, η*_p_*^2^ = 0.23; *F* < 1.23, *p* > 0.28 in all other areas). Finally, as in Experiment 1, no significant difference was found between the baselines of high- and low-load conditions (mean difference ≤ 0.0032 μm; all *p*_FDR_ > 0.131).

To assess the impact of perceptual load on attended processing, we analyzed the effect of load on the oxCCO signal related to the attended stream in the distractor-absent (target only) conditions in all areas. As can be seen in Figure 3*A*, *C*, the mean oxCCO response to the targets (in the distractor-absent conditions) was increased under high perceptual load, and this reached significance in left BA18 (*t*_(16)_ = 2.98, *p*_FDR_ = 0.022, *d* = 0.72) and BA17 (*t*_(16)_ = 3.49, *p*_FDR_ < 0.015, *d* = 0.85). Similar trends in the other BAs failed to reach significance (all *t* < 1.63, *p*_FDR_ > 0.169).

**Figure 4.**
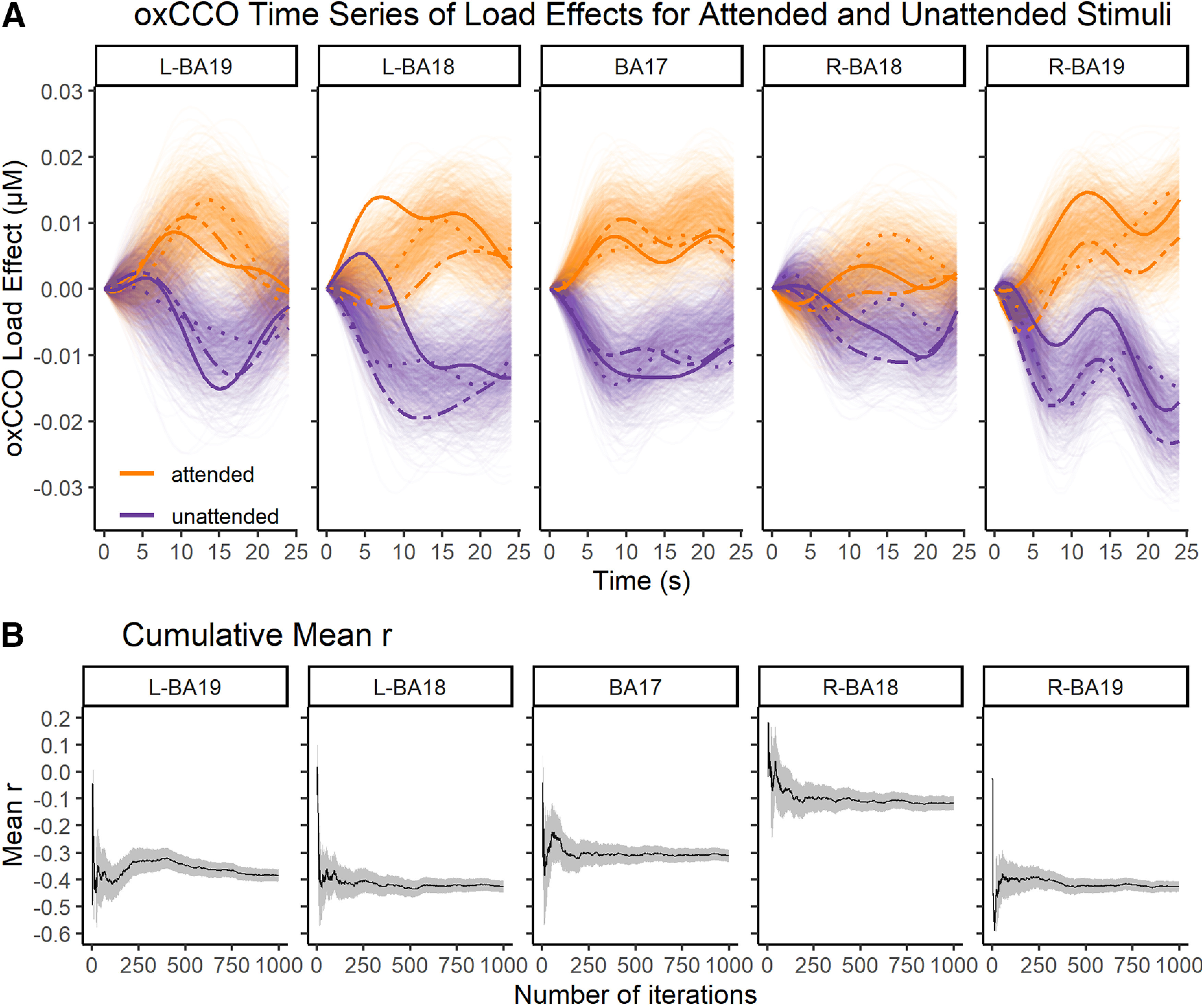
Time course of the load effects on oxCCO signal associated with attended and unattended stimulus processing. ***A***, Time series of the load effects (high – low) on the oxCCO signal underlying attended and unattended processing are shown for each iteration of the data-splitting procedure. Bold lines with matching line types indicate three specific iteration instances of time-series pairs for which the *r* value was closest to the mean *r* value across all conditions, shown for illustrative purposes. ***B***, Cumulative mean *r* values across 1000 iterations of the random sample splitting procedure, which represents the correlation between the load effects on attended and unattended processing. Gray, shaded error bars represent 95% CIs. The mean *r* value can be seen to stabilize on the resultant mean after ∼200 iterations in all areas. Moreover, the narrow 95% CIs (already found at ∼500 iterations) indicate that the resultant mean is a reliable representation of the correlation between the two time series.

In addition, we assessed the temporal correlation between the effects of load on oxCCO levels related to attended processing and the load effects on oxCCO levels related to unattended processing during the 25 s task period. The results are shown in Figure 4. As can be seen in the figure, the temporal (second-by-second) patterns of the effects of load on attended and unattended signals were negatively correlated in all areas. A random permutation test (for details on this analysis, see Materials and Methods) showed that all these correlations were significant (left BA19: mean *r* = −0.38, *p*_FDR_ < 0.001; left BA18: mean *r* = −0.43, *p*_FDR_ < 0.001; BA17: mean *r* = −0.31, *p*_FDR_ < 0.001; right BA18: mean *r* = −0.12, *p*_FDR_ < 0.001; right BA19: mean *r* = −0.43, *p*_FDR_ < 0.001). These findings indicate a “push-pull” trade-off relationship between metabolism levels related to attended and unattended processing as a function of perceptual load in the task.

Finally, the hypothesis of constant energy supply regardless of mental task demand (i.e., perceptual load) receives additional support when oxCCO levels are measured while both attended and unattended stimuli are present. As shown in Figure 3*A* (distractor-present conditions), metabolism levels remain constant across the low-load and high-load conditions in all five regions when thus measured (all *p*_FDR_ > 0.440). This is explained by a spillover to the processing of the distractor in the low-load conditions, but not high-load conditions, which are likely to approach the set energy limit already with the attended processing alone.

## Discussion

The present results provide support for our proposed cellular metabolism account for perceptual capacity limits and the role of attention in perception. Specifically, the findings established attention-dependent modulation of cellular metabolism levels in visual cortex in line with the changes in perceptual load levels in the task. Higher perceptual load in the task was associated with increased cellular metabolism levels related to attended processing and reduced levels related to unattended processing in the form of a direct resource trade-off. This “push-pull” relationship is further supported by a negative correlation between the temporal pattern of load effects on metabolism levels associated with attended versus unattended processing. Perceptual capacity limits and the consequent effects of reduced unattended processing in conditions of high perceptual load may therefore be attributed to a shortage in cellular metabolism for processing stimuli outside the focus of attention.

Our account offers a neurobiological explanation of the large body of studies showing attentional modulations of task performance and perception as well as the related cortical activity caused by high perceptual load in the task. The previous findings have been obtained with a variety of behavioral tasks and attentional manipulations (Simons and Chabris, 1999; Carmel et al., 2007; Cartwright-Finch and Lavie, 2007; Macdonald and Lavie, 2008; Murphy and Greene, 2016) and in functional imaging studies (Rees et al., 1997; Handy and Mangun, 2000; Handy et al., 2001; Pinsk et al., 2004; Yi et al., 2004; S. Schwartz et al., 2005; Nagamatsu et al., 2011; Parks et al., 2013; Molloy et al., 2015; Torralbo et al., 2016). The present results suggest that these well-established modulations can be explained by changes in cellular metabolism levels in visual cortex.

Importantly, oxCCO levels provide a direct, intracellular measure of neural metabolism because of the CCO enzyme's integral role in cellular oxygen metabolism (as the final electron acceptor in the respiratory electron transport chain of the mitochondria). In contrast, the hemodynamic response measured with fMRI cannot be used to directly infer the level of underlying cellular metabolism, despite being correlated with it (Logothetis, 2008). Specifically, the level of HHb in local blood vessels, underlying the BOLD response, is not only influenced by the level of cellular oxygen metabolism, but in even greater measure by the rate of cerebral blood flow (Fox and Raichle, 1986; Buxton and Frank, 1997). While oxygen metabolism is driven by the energy demand following neural activity, increases in cerebral blood flow are thought to be driven primarily by the presence of the excitatory neurotransmitter glutamate; these two processes can therefore be considered as related, but operating in parallel (Attwell and Iadecola, 2002). Moreover, the ill-understood, variable coupling of the two over space and time further complicates any inference about oxygen metabolism (Logothetis, 2008; Lindquist et al., 2009).

The present findings also lend support to the influential (e.g., Raichle and Gusnard, 2002; Lennie, 2003; Carrasco, 2011; Lauritzen et al., 2012) notion that overall cerebral metabolism remains constant regardless of mental task demand (Sokoloff et al., 1955), and despite the high energetic cost of neural firing (Attwell and Laughlin, 2001; Lennie, 2003). While much theoretical and modeling work presumed this notion, subsequent empirical evidence for this claim has been scarce. In a repeatedly cited study, Sokoloff et al., (1955; see also Clarke and Sokoloff, 1999) used a nitrous oxide technique as a measure of whole-brain cerebral metabolic rate (CMRO_2_). Overall CMRO_2_ during rest did not significantly differ from overall CMRO_2_ during a mental (arithmetic) task condition. While often cited as evidence for a constant and therefore limited metabolic energy capacity of the human brain, this conclusion rests on a null result. Here, we similarly report constant oxCCO levels regardless of mental task demand (i.e., load) when these are measured as summed activity across both attended and unattended processing. This finding was expected based on load theory predictions that spare capacity spills over to the processing of unattended stimuli in low perceptual load conditions, so that the overall level of metabolism remains the same as in high-load conditions (when more capacity is exhausted by attended processing). Thus, just the distribution between attended and unattended processing differs between load conditions. Importantly, we additionally report findings that positively demonstrate this trade-off effect of mental processing demand on cerebral metabolism levels related to attended versus unattended processes. This finding, alongside the temporally specific correlation of load effects, directly supports the commonly made assertion that limited metabolic resources are redistributed to flexibly adapt to mental task demands (Raichle et al., 2001; Carrasco, 2011), highlighting the role of attention in control over the metabolic resource allocation. We suggest that the observed trade-off is the result of an attention mechanism that serves to balance metabolic supply and demand across the brain, in line with current processing priorities.

Our results fit with the well-established findings that increases in cellular metabolism during enhanced neural firing are primarily needed for the energetically expensive process of restoring ion gradients after depolarization of the cell membrane. The observed pattern of responses therefore reflects changes in the number of action potentials sent within the area of measurement. However, a considerable contribution to the signal is likely also made by a change in the number of incoming signals (i.e., postsynaptic potentials). The integration of postsynaptic potentials has been shown to require more metabolism than firing action potentials (W. J. Schwartz et al., 1979), suggesting that this may contribute more to our observed effects than just action potential generation. Since attention is known to involve extensive feedback connections between higher-level areas (frontal and parietal cortices) and sensory cortices (Dehaene et al., 1998; Silvanto et al., 2009; Wei et al., 2013; Torralbo et al., 2016), incoming signals from these areas likely play a role in the changes in metabolic patterns observed here in visual cortex, in addition to incoming signals from lower-level areas and local connections.

It is also important to consider how the present results relate to previous behavioral findings. The perceptual load manipulation used in our study is well established (S. Schwartz et al., 2005; Bahrami et al., 2007; Carmel et al., 2011; Ohta et al., 2012) and known to converge with other manipulations of perceptual load (e.g., spatial visual search, set-size manipulations) to demonstrate reduced unattended processing, leading to “inattentional blindness.” Importantly, these effects are found with both implicit measures of unattended processing (e.g., neuroimaging, distractor effects on reaction time), which are collected for concurrent attended and unattended processing, as here, and explicit detection sensitivity measures, including measures of detection responses made immediately on appearance (e.g., Macdonald and Lavie, 2008; Lavie et al., 2014), which rule out alternative accounts attributing inattentional blindness to “inattentional amnesia.” The convergence of findings suggests that alternative accounts of the present findings in terms of task-specific factors are unlikely. For example, while the current task included an extra feature to be remembered in high load (low load: upright or inverted red cross; high load: upright purple or inverted blue cross), and thus perhaps increased visual short-term memory load, other feature-versus-conjunction load manipulations that equated the number of features have found consistent results (Wojciulik and Kanwisher, 1999; Stolte et al., 2014). Moreover, visual short-term memory load is known to affect unattended processing in the same way as perceptual load, unlike other types of WM load that tap more into cognitive control (Lavie et al., 2004; Konstantinou et al., 2012, 2014; Konstantinou and Lavie, 2013); and since visual short-term memory has been shown to recruit sensory cortices (e.g., Pasternak and Greenlee, 2005), the explanation of our results based on a metabolic resource trade-off in visual cortex still applies.

Finally, our metabolism trade-off account opens up many novel questions for future research regarding the nature of capacity limits, for instance, regarding the spatial scale of the trade-off and whether it extends to multimodal processes. Furthermore, while we demonstrated that attention can lead to the flexible redistribution of metabolism based on task demand, this may also occur in spatial cueing or feature-based attention paradigms. Future research should investigate whether such manipulations of attentional engagement lead to similar metabolism trade-offs.

In conclusion, the concept of a mental processing resource with limited capacity has dominated attention research for decades (Navon and Gopher, 1979; Wickens et al., 1984; Lavie et al., 2014; Molloy et al., 2019); however, its relationship to the biochemical resources mediating neural activity remained unclear. Here, we provide evidence for our proposal that this frequently theorized, capacity-limited, mental resource corresponds to limited cellular metabolic energy across the brain. Our findings demonstrate that the level of perceptual load in the task modulates the impact of attention on cellular metabolism levels in visual cortex regions related to stimulus perception. Increased perceptual load leads to increased levels of metabolism underlying attended processing at the expense of unattended processing, thus explaining phenomena of inattentional blindness. Moreover, this resource trade-off supports the notion that the overall cerebral metabolic energy supply remains constant regardless of mental task demand, by demonstrating how increases in processing demand, and the associated demand for metabolic energy, are balanced out by equivalent, parallel decreases in metabolism, to maintain a constant level overall.
